# Effects of Hydrophobic Phase Properties on Controlling Nanoparticle Jamming at Oil/Water and Air/Water Interfaces

**DOI:** 10.1021/acs.langmuir.5c01946

**Published:** 2025-08-07

**Authors:** Olivia M. Haider, Lynn M. Walker

**Affiliations:** † Department of Chemical Engineering, 6612 Carnegie Mellon University, Pittsburgh, Pennsylvania 15213, United States; ‡ Department of Chemical Engineering and Materials Science, University of Minnesota, Minneapolis, Minnesota 55455, United States

## Abstract

Fluid/fluid interfaces stabilized with strongly adsorbed solid nanoparticles are implemented in industries including cosmetics, pharmaceuticals, and food science. Solid particles at the interface result in complex interfacial mechanics, which are highly dependent on interfacial particle behavior and bulk properties of both fluid phases. Many interfacial studies have been conducted characterizing the effects of the aqueous fluid properties such as particle chemistry, pH, temperature, salinity, and the impact of surfactant and other additives on interfacial mechanics and adsorption behavior. However, the role of the hydrophobic phase on interfacial stability, as well as the adsorption and organization of interfacial material, is less understood. In this work, mechanical properties of particle-laden interfaces are characterized at oil/water and air/water interfaces to determine the impact of the hydrophobic fluid on particle jamming at the fluid/fluid interface. A model aqueous phase containing CTAB-SiO_2_ (cationic surfactant-anionic nanoparticle) complexes is used to deliver particles to air/water, dodecane/water, and silicone oil/water interfaces using a fixed adsorption protocol. Adsorption dynamics and interfacial rheology are measured on microscale using a microtensiometer platform. Results show that under the same aqueous conditions, the hydrophobic phase impacts the effective areal coverage of particles at the interface. When subjected to nonlinear compression cycles, interfacial jamming is impacted by particle wettability and electrostatic interactions between adsorbed particles. These findings suggest that the nonpolar phase has a significant impact on the lateral interactions between particles at the interface. This work highlights how changes to the hydrophobic fluid introduce additional complexities to the interfacial properties and further the understanding of solid particle interactions at different fluid interfaces for controlled emulsion design.

## Graphical abstract

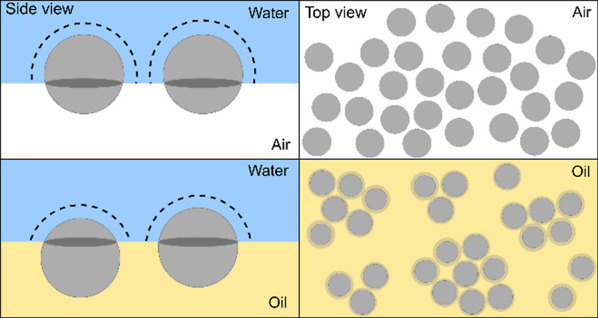

## Introduction

Strongly adsorbed solid particles, used in place of surfactants and other chemical emulsifiers in emulsions and foams, significantly change the mechanical properties of the fluid/fluid interface and can hinder both coalescence and interfacial deformation.^1–8^ Enhanced interfacial stability of nanoparticle-coated interfaces in these systems (referred to as “Pickering” systems) is attributed to the observed significant bulk stability in emulsions and foams.^1–3,9,10^ There is increasing interest in utilizing solid particle stabilizers in a variety of industries where controlling emulsion stability is critical: oil-spill remediation,^11^ product design in fields such as food science,^12^ pharmaceutical development,^3^ cosmetics and personal care,^13^ the design of high internal phase emulsions (HIPEs),^14^ and others. When the packing fraction of particles trapped on a fluid/fluid interface reaches a maximum, the system achieves a two-dimensional jammed state, fundamentally changing the interfacial mechanics^8,15–18^ and driving macroscopic stability. Because properties of the fluid/fluid interface impact bulk mechanical properties, understanding particle behavior and interfacial properties of systems in this jammed state is key to the controlled design of soft, multiphase materials.

Nanoparticles pinned to fluid/fluid interfaces are constrained by a strong adsorption or pinning energy (*E*) given in eq 1

1 $$E=\pi R^{2}\gamma {\left(1\pm \cos\theta \right)}^{2}$$

which is defined by the three-phase contact angle (θ), particle radius (*R*), and interfacial tension between the two fluid phases (γ_ow_, or γ_aw_).^3,6,7,19–21^ The nature of the two fluid phases contribute to this pinning energy in two ways, directly through the interfacial tension (air/water, γ_aw_ or oil/water, γ_ow_) and then also though the nature of the wetting, or the contact angle set by the surface energy between the fluids and the particle surface (denoted as γ_ap_ or γ_op_). The three-point contact line governs the wetting conditions, where the particle sits at the interface. The wetted cross-sectional area (*A*_p_) of a spherical particle is related to the contact angle (θ) by

2 $$A_{p}={\pi \left(r\cdot \cos\left(\theta -90\right)\right)}^{2}$$

where *r* is the radius of the particle. The contact line and adsorption energy controls the strength of particle adsorption at the interface and the ability for the particles to dewet due to interfacial processing. The adsorption energy is greatest at a contact angle of 90°.^19^ At larger angles, the particle prefers the oil phase, and smaller angles indicate stronger attraction to the aqueous phase.^9,22^ At the air/water interface, contact angles of bare SiO_2_ particles are estimated to be ∼20–37°.^2^ For oil–water interfaces, particles tend to sit farther into the oil phase, with typical contact angles of θ > 90°. Interfacial tension varies with the type of hydrophobic fluid used, where γ_aw_ = 72 mN/m for a clean air/water interface, and common alkane water systems have γ_ow_ ∼ 30–50 mN/m.^24^ For a 20 nm nanoparticle, the pinning energy is typically 100–1000 times kT at room temperature, making the adsorption practically irreversible.

To optimize partial wetting conditions and increase the adsorption energy of the particles, the three-phase contact angle can be modified with the addition of surfactants, surface chemistry modifications, particle size and shape, and the wetting behavior of both the water and oil.^3,6,11,21,23,25,26^ For particle-loaded interfaces, as the distances between particles on the interface are short, electrostatic and attractive colloidal forces contribute to interfacial mechanics and lateral particle stability on the interface.^17,27^ These forces are directly related to the interfacial properties of the two fluid phases and the solid particle surface.^4,28^

The nature of the bulk fluid phases defines the properties of the solid particles at interfaces. Many interfacial studies quantify the effects of the aqueous phase, investigating the impact of system parameters such as temperature, pH,^29,30^ particle size and surface properties,^5,19,31–33^ and the incorporation of additives such as surfactants.^5,6,9,11,19,31,32^ The nature of the hydrophobic fluid, however, significantly impacts the behavior of these interfaces, but the effects of the hydrophobic phase on interfacial stability and particle jamming are less understood. Properties of the hydrophobic phase are shown to impact the three-phase contact angle and the energy associated with pinning the particles at the interface as both are governed by the interfacial tension between the two bulk phases. Interfacial studies have noted the oil chemistry of various alkanes and triglycerides impacts surfactant adsorption,^34–38^ nanoparticle coverage,^36,39,40^ and protein morphology at the interface.^29,30,36,41–45^ Hydrophobicity and oil polarity are shown to affect the packing, morphology, and surface coverage of the adsorbed molecular species at the interface. For example, the surface concentration of charged surfactants increases with increasing oil polarity due to additional interactions between the oil and water at the surface.^34^

Previous work done in our group developed methods for processing and characterizing multicomponent, particle-laden interfaces.^2,46–48^ It was shown that the rate of transport of complexes to the interface is dependent on the complex size (diffusivity) rather than the surfactant tail length. However, the nature of the surfactant governs the steady-state interfacial tension and amount of material adsorbed to the interface. Additionally, the behavior of the nanoparticles pinned at the interface governs the interfacial mechanical properties. Results from these studies characterized the interfacial properties at air/water^2,47,49^ and oil/water^46,48^ interfaces while demonstrating processing methods that can control the mechanical properties of bubbles and droplets with particle-laden interfaces.

In this work, we determine key system parameters and interfacial processing methods to control the properties of particle-laden interfaces. Effects of hydrophobic fluid on adsorption dynamics, the amount of adsorbed material, and interparticle interactions are shown to significantly impact the characteristics of particle jamming at the interface. Here, we will use a model surfactant–particle complex system and consider two chemically different oils to determine the impact of the hydrophobic phase on particle adsorption and interfacial mechanics.

## Materials and Methods

Throughout this study, a dispersion of surfactant-nanoparticle complexes, studied previously at air/water interfaces,^2^ is used as a model aqueous system. A 34 wt % dispersion of Ludox TMA SiO_2_ nanoparticles is purchased from Sigma-Aldrich (St. Louis, MO) and used as received. The surfactant hexadecyl trimethylammonium bromide (CTAB) from Sigma (St. Louis, MO) is purchased at 99% purity. To remove impurities, the surfactant is recrystallized from ethanol twice before use. Sodium chloride (NaCl) is purchased at 99% purity from VWR (Batavia, IL) and is baked at 400 °C for 5 h, removing hydrates and impurities. Deionized (DI) water, prepared with a Millipore filtration system, has a set resistivity of 18.2 MΩ·cm. The oils used for this work are *n*-dodecane and 96 mPa·s silicone oil (Sigma, St. Louis, MO). Oils are passed through a column of alumina powder prior to use to remove surface-active impurities.

### Solution Preparation and Characterization

In this work, the concentration of SiO_2_ particles in the aqueous phase is constant at 10 wt % with different ratios of SiO_2_ to surfactant. At surfactant concentrations of 0.1 and 0.3 mM CTAB, this results in a ratio of approximately 8 or 24 surfactant molecules per particle on average, respectively. These ratios were determined using a particle diameter of 22 nm and density of 2.2 g/cm^3^, as reported by the manufacturer. Zeta potential measurements for dispersions of 1 wt % Ludox TMA at varied concentrations of CTAB in 10 mM NaCl are used to determine the impact of the CTAB to particle ratio on net complex charge. Concentrations of 0.03 mM CTAB or lower do show a significant increase in the zeta potential compared to that of bare silica, as shown in Figure S1 of the Supporting Information. Additionally, no indication of visible sedimentation or flocculation at these low concentrations is observed. For these interfacial studies, equivalent ratios of CTAB to silica in dispersions with 10 wt % nanoparticles are used. The final compositions of the aqueous samples are either 0.1 mM CTAB or 0.3 mM CTAB with 10 wt % silica particles in 10 mM NaCl. To minimize the potential of aggregation, the solution was prepared by adding the 34 wt % Ludox dispersion dropwise to the CTAB and NaCl mixture under sonication. After the addition of nanoparticles, solutions are sonicated further for 30 min and then aged under ambient conditions for 24 h. Samples are stored for at most 48 h, minimizing uncertainty due to the potential aggregation of silica.

### Interfacial Measurements

Interfacial measurements are taken during the development and processing of particle-coated interfaces using a microtensiometer platform, shown in Figure 1a and detailed in previous studies.^47,49–53^ Briefly, a capillary filled with the hydrophobic fluid is connected in-line with a pressure transducer (Omegadyne INC., Sunbury, Ohio) and submerged into a semi-infinite reservoir (∼3 mL) containing the aqueous fluid. The microtensiometer device consists of a 3-D printed cell made from Delrin (Polyoxymethylene). Glass capillaries are pulled to a tip radius of 36–45 μm using custom settings on a PMP-102 micropipet puller (MicroData Instrument, INC., South Plainfield, NJ). The interior of the capillary is acid washed with 30% sulfuric acid in DI water and then coated with hydrophobic material from Dynasylan SIVO CLEAR (Evonik Industries, Essen, Germany) to ensure the three-phase contact line remains pinned at the tip of the capillary. Capillaries are rinsed with ethanol after coating and then baked at 60 °C for 30 min prior to use. Interfaces are imaged using a Nikon T-300 inverted light microscope and digital camera (Point Gray Flea).

**Figure 1:**
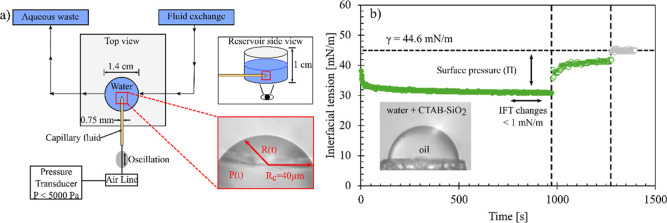
(a) Schematic of the microtensiometer platform. A capillary is connected to an in-line pressure transducer with a controlled back pressure and submerged in a reservoir containing the aqueous phase. Inlet and outlet ports connected to the bulk reservoir allow for controlled fluid exchange. (Top right) Reservoir side view. (Bottom right) The interface forming a hemispherical cap pinned at the capillary tip with a capillary radius (*R*_c_) of approximately 40 μm. (b) A representative experiment demonstrating the interfacial processing procedure, with interfacial tension of a silicone oil/water interface shown as a function of time during the adsorption of CTAB-SiO_2_ complexes (filled circles), during a bulk reservoir exchange with DI water (open circles), and after the formation of new interface (gray circles). An image of the interface at the capillary tip is shown, with silicone oil is inside the capillary, and an outer aqueous phase of CTAB-SiO_2_ complexes. Here, a solution of 0.3 mM CTAB, 10 wt % Ludox TMA nanoparticles, and 10 mM NaCl was used as the aqueous phase.

The radius of the interface is measured using routines written in LABVIEW (National Instruments). From the measured radius of curvature and pressure jump across the interface, the interfacial tension is determined using the Young–Laplace equation

3 $$\gamma \left(t\right)=\frac{\left(P\left(t\right)-P_{h}\right)R\left(t\right)}{2}$$

where *P*(*t*) is the pressure within the capillary fluid measured with time, *P*_h_ is the hydrostatic pressure above the capillary tip, *R*(*t*) is the radius of the interface as a function of time, and γ­(*t*) is the calculated dynamic interfacial tension. The hydrostatic pressure is constant over the experiment duration and is determined by measuring the interfacial tension at different values of pressure and radius for a clean o/w interface. To demonstrate the experimental method of processing particle-laden interfaces, Figure 1b shows interfacial tension measurements at a silicone oil/water interface. A clean interface is generated at *t* = 0 by ejecting an existing drop of oil from the capillary tip with a spike in pressure within the capillary fluid. Interfacial tension is measured over an adsorption time of 1000 s. After the adsorption of the CTAB-SiO_2_ complexes to the interface, the bulk fluid in the reservoir is exchanged with fresh DI water. In between the adsorption and reservoir exchange steps, the interfacial properties of particle-coated interfaces are characterized.

To characterize the mechanics during large compression cycles, a syringe pump (Braintree Scientific, Inc.) connected to the air line is used to decrease the pressure within the capillary fluid, subjecting the interface to compressions with up to a 50% change in the surface area. From the interfacial tension response, the Gibbs modulus is used to evaluate the interfacial behavior during these nonlinear compression cycles and is defined as

4 $$E_{G}=\frac{d\gamma }{d\;\ln A}$$

The interfacial area (*A*) is that of a hemispherical cap and is calculated as

5 $$A=2\pi R\left(t\right)\left(R\left(t\right)-\sqrt{R{\left(t\right)}^{2}-R_{C}^{2}}\right)$$

where *R*(*t*) is the radius of curvature measured over time and *R*_C_ is the radius of the capillary. The measurement of uncertainty of the radial measurement is 0.1 μm, which results in the maximum measurable modulus on the order of 1000 mN/m.^47^ The errors in the pressure and radius measurements are 10 Pa and 0.1 μm, respectively, resulting in an assumed error of ±1 mN/m of the instrument.

## Results and Discussion

Dynamic interfacial tension and surface pressure measurements over a 1000 s adsorption time are shown in Figure 2a and b, respectively. Adsorption dynamics measure the delivery of CTAB-SiO_2_ complexes using three different hydrophobic phases: air (squares), *n*-dodecane (diamonds), and 96 mPa·s of silicone oil (circles). Dashed lines show the measured clean interfacial tension values (γ_0_) for each interface. Values of γ_0_ in DI water are 72.0, 52.5, and 44.6 mN/m for air/water, dodecane/water, and silicone oil/water, respectively. At a concentration of 10 mM NaCl, changes on the interfacial tension due to the salt are assumed to be less than 1 mN/m.^54^ Here, *t* = 0 corresponds to the formation of a new interface, and the interfacial tension decreases with time as the surfactant-nanoparticle complexes adsorb to the interface. Decay of flow due to the formation of the clean interface (bubble) is within 1 s for a/w interfaces and is ∼10 s for o/w interfaces, which are not noticeable on these adsorption timescales.^46,52^ The initial decrease in interfacial tension at each interface is not captured in these measurements due to rapid initial adsorption of CTAB-SiO_2_ complexes.

**Figure 2:**
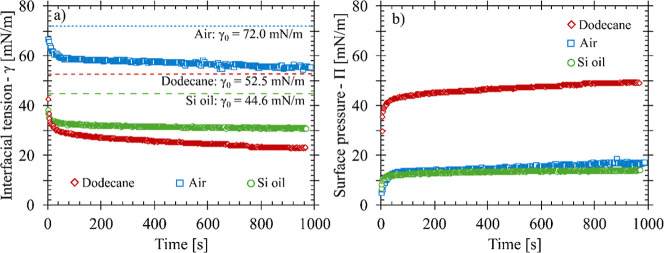
(a) Interfacial tension and (b) surface pressure measurements as a function of time for CTAB-SiO_2_ complexes at the air/water interface (squares), silicone oil/water interface (circles), and *n*-dodecane/water interface (diamonds). Dotted lines on (a) correspond to the respective clean interfacial tension values for each system. The concentration of CTAB used for each system was 0.3 mM.

Equilibrium is not observed at the interfaces measured here; instead, a system is assumed to be at steady state when there is less than a 1 mN/m change in the interfacial tension over a 1000 s adsorption time. As this is observed for both a/w and o/w systems, an arbitrary adsorption time of 1000 s is used to characterize the interfacial properties. This approach is consistent with work in similar systems and in controlled interfacial studies where all the material delivered from the interfaces is from the aqueous phase.^2,49^ The surface pressure (Π) of each interface is determined from the dynamic measurements and clean interfacial tension values (γ_0_) of each interface as

6 $$\Pi =\gamma_{0}-\gamma$$

This allows for the surface properties to be compared between systems with different values of γ_0_. Table 1 shows the interfacial tension and surface pressure measurements for air/water, dodecane/water, and silicone oil/water interfaces, reported with the standard error between experimental replicates. Adsorption experiments were repeated a minimum of four times except for Π_rinse_ for air and dodecane, which have one replicate. At the air/water interface, the interfacial tension after 1000 s, γ_1000_, is 58.1 ± 0.8 mN/m, while the interfacial tension at the oil/water interfaces are at 22.3 ± 0.42 for dodecane and 30.8 ± 0.13 for silicone oil.

**Table 1:** Interfacial Tension Values for the Air, *n*-Dodecane, and Silicone Oil Systems at a Clean Interface (γ_0_) and After a 1000 s Adsorption Time of the CTAB-SiO_2_ Complexes (γ_1000_)­^a^

units: [mN/m]	air	*n*-dodecane	silicone oil
γ_0_	72.0	52.5	44.7
γ_1000_	58.1 ± 0.8	22.3 ± 0.42	30.8 ± 0.13
Π_1000_	13.9 ± 0.8	30.2 ± 0.42	13.9 ± 0.13
Π_rinse_	1.33	7.04	3.0 ± 0.10

a Surface pressures after 1000 s adsorption time (Π_1000_) and after a bulk fluid exchange (Π_rinse_) are given for each system. The standard error is reported for systems with four or more experimental replicates.

The oil polarity and chain length are important factors in the steady-state surface pressure (Π_inf_) and molecular arrangement of surfactants at the interface at the interface,^34,36^ so we assume the surface coverage is driven by CTAB adsorption and make use of data measured at different oil interfaces.^55^ The interfacial tension values measured for CTAB-SiO_2_ complexes at the air/water and dodecane/water interfaces are comparable to those of pure CTAB, which were measured to be approximately 54 mN/m for air/water and 19 mN/m for dodecane/water at equilibrium. Additionally, the surface excess concentration (Γ) was evaluated using the Gibbs equation and interfacial tension measurements, and results showed similar values of Γ in air/water and dodecane/water systems at a concentration of 0.3 mM CTAB.^55^ This suggests that approximately the same amount of material is delivered to each interface. With an average ratio of 24 molecules of CTAB per SiO_2_ particle, and assuming all the CTAB within the complexes is available to interact with the interface, it is assumed that the number of silica particles delivered to both air/water and dodecane/water interfaces is effectively the same at a fixed concentration of CTAB. Additionally, the delivery of particles to the interface is driven by the surface-active species, which is consistent with previous findings,^2^ using 0.1 mM C_*n*_TAB surfactants to deliver SiO_2_ particles to the air/water interface.

To investigate the irreversibility of adsorbed particles, a bulk fluid exchange of aqueous fluid is used to remove bulk CTAB-SiO_2_ complexes from the reservoir and replace the dispersion with clean DI water. The duration of this “rinsing” process is a minimum of 30 times the residence time (τ_R_) of the bulk reservoir. In this time, surface pressure decreases as CTAB leaves the interface. After the mixture is rinsed, surface pressure at the air/water interface decreases to 1.33 mN/m, which is near the measurement resolution of the microtensiometer. At the oil/water interfaces, surface pressure after the bulk exchange (Π_rinse_) is measured to be 7.04 mN/m for dodecane and 3.00 mN/m for silicone oil. While the surface pressure decreases due to surfactant desorbing from the interface, some molecules likely remain after the reservoir exchange process along with the majority of the nanoparticles. This is evidenced by the measurable surface pressures after the bulk fluid exchange (Π_rinse_) as the nanoparticles themselves are not expected to contribute to the surface pressure once adsorbed.^56^ Although the surface-active species is the main contributor to the measured interfacial tension, these measurements alone are insufficient to describe the particle coverage and jamming behavior at the interface.

The effect of concentration on interfacial jamming behavior is analyzed for air–water interfaces after 1000 s adsorption of CTAB-SiO_2_ complexes and after a bulk reservoir exchange to pure water. Figure 3 shows the results for large compressions of air/water interfaces and are used as a qualitative characterization of interfacial behavior. Measurements for a single compression after the adsorption step are shown with closed symbols, while open symbols correspond to the compression after the reservoir exchange described above and in Figure 1. These measurements are plotted as a function of the fractional area change of the interface (Δ*A*/*A*_0_) as the absolute surface area varies between experiments due to small differences in capillary radii and other experimental conditions.

**Figure 3:**
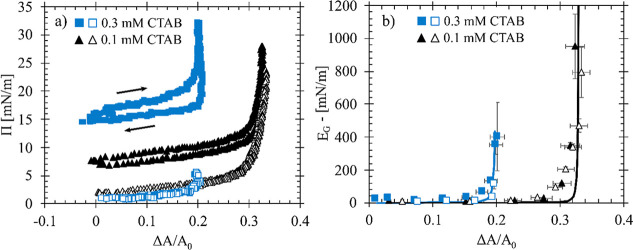
(a) Surface pressure and (b) Gibbs modulus as a function of fractional area change for air/water interfaces exposed to 0.1 mM CTAB with 10 wt % SiO_2_ in 10 mM NaCl (triangles) and 0.3 mM CTAB with 10 wt % SiO_2_ in 10 mM NaCl (squares). Closed symbols are for systems with the dispersion present in the reservoir and open symbols are compression of interfaces after a bulk fluid exchange of DI water.

Figure 3a shows surface pressure measurements during the compression and expansion of an air/water interface exposed to 0.1 mM CTAB (triangles), measured from a previous study,^2^ and 0.3 mM CTAB in the aqueous phase (squares), measured here. Initially, the surface pressure slowly increases as the interface is compressed and Δ*A*/*A*_0_ increases. Once a critical area is reached, the surface pressure increases sharply and is resistant to any further compression. As the interface is decompressed, hysteresis is observed, with the surface pressure returning to the initial value. This suggests that the compression is reversible, and the interface has not been permanently impacted by the nonlinear compression. The magnitude of the surface pressure increases with increased concentration of CTAB. This result follows the behavior of surfactant adsorption measurements below the CMC.^57^ This impact of CTAB concentration and change in the ratio of surfactant to particles follows an expected trend in which higher bulk concentrations lead to faster adsorption dynamics and higher surface concentrations of adsorbed surfactant. Thus, increasing the concentration of CTAB in the aqueous solution leads to more material at the interface, demonstrating how the overall concentration of the surfactant can control particle delivery.

To probe the interfacial mechanics at different stages of interfacial processing, the local slope of each compression curve is taken as an estimate of the Gibbs modulus (*E*_G_) shown in Figure 3b. Assuming that the SiO_2_ particles are irreversibly adsorbed at the interface, the surface concentration of particles increases with decreasing surface area until there is a rapid increase in surface pressure. Here, there is a sharp increase in the *E*_G_, and the interface appears to stiffen with small changes in the compression. This response is indicative of interfacial jamming as particles reach maximum packing on the surface. Because solid particles are assumed to be irreversibly adsorbed, the fractional area change is equivalent to the ratio of surface concentrations of particles at the interface. This is given as

7 $$\xi =\frac{\Delta A}{A_{0}}=1-\frac{\phi_{0}}{\phi }$$

where ϕ_0_ and *A*_0_ are the initial surface concentration and surface area of the interface prior to the compression, Δ*A* is the change in surface area due to the compression, and ϕ is the particle fraction of a compressed interface. At the point of interfacial jamming, the value of ϕ is assumed to be ϕ_c_ = 0.86, which is the maximum packing fraction of a 2-dimensional random close packed system with hard sphere interactions.^6^ We define a critical area change

8 $$\xi_{c}=\frac{{\Delta A}_{c}}{A_{0}}$$

as the area change at which the modulus sharply increases, and the interface is resistant to any further compression. Here, Δ*A*_c_ is the overall surface area change required to jam the interface. In all cases, the modulus is initially low, on the order of 10 mN/m, and increases sharply when the interface is compressed and the areal change approaches ξ_c_. At this point, the particles at the interface are assumed to reach a maximum packing, preventing the interface from compressing further. Results from Figure 3b show that ξ_c_ decreases with increasing CTAB concentration, which suggests an increase in the number of particles delivered to the interface due to the surfactant concentration. Because the amount of material at the interface is assumed to be driven by the adsorption of the surface-active species, these results demonstrate that the particle concentration at the interface is controlled by adjusting the bulk concentration of surfactant. The Gibbs modulus behavior of air/water interfaces after the bulk fluid exchange are shown as the open symbols in Figure 3b. Results here show that ξ_c_ is the same for air–water interfaces exposed to either bulk particle complexes or clean DI water. This suggests that the particles at the interface are not removed during the reservoir exchange process despite the desorption of CTAB molecules; this agrees with previous work^2^ and demonstrates the irreversibility of these strongly absorbed nanoparticles.

The impact of the hydrophobic phase on interfacial jamming properties and particle coverage is investigated further using air, *n*-dodecane, and silicone oil as the hydrophobic fluids. The aqueous system used for these experiments consists of 0.3 mM CTAB and 10 wt % SiO_2_ in 10 mM NaCl. Figure 4a shows the same compression cycles for the air/water interface (shown in the blue squares) as Figure 3, replotted here along with compression cycles of dodecane/water (red diamonds) and silicone oil/water (green circles) interfaces. Figure 4b shows compression and expansion cycles after a bulk fluid exchange replaces the reservoir fluid with fresh DI water for each system. To minimize changes to the interfacial structure due to processing, only one compression experiment was performed on a single interface.

**Figure 4:**
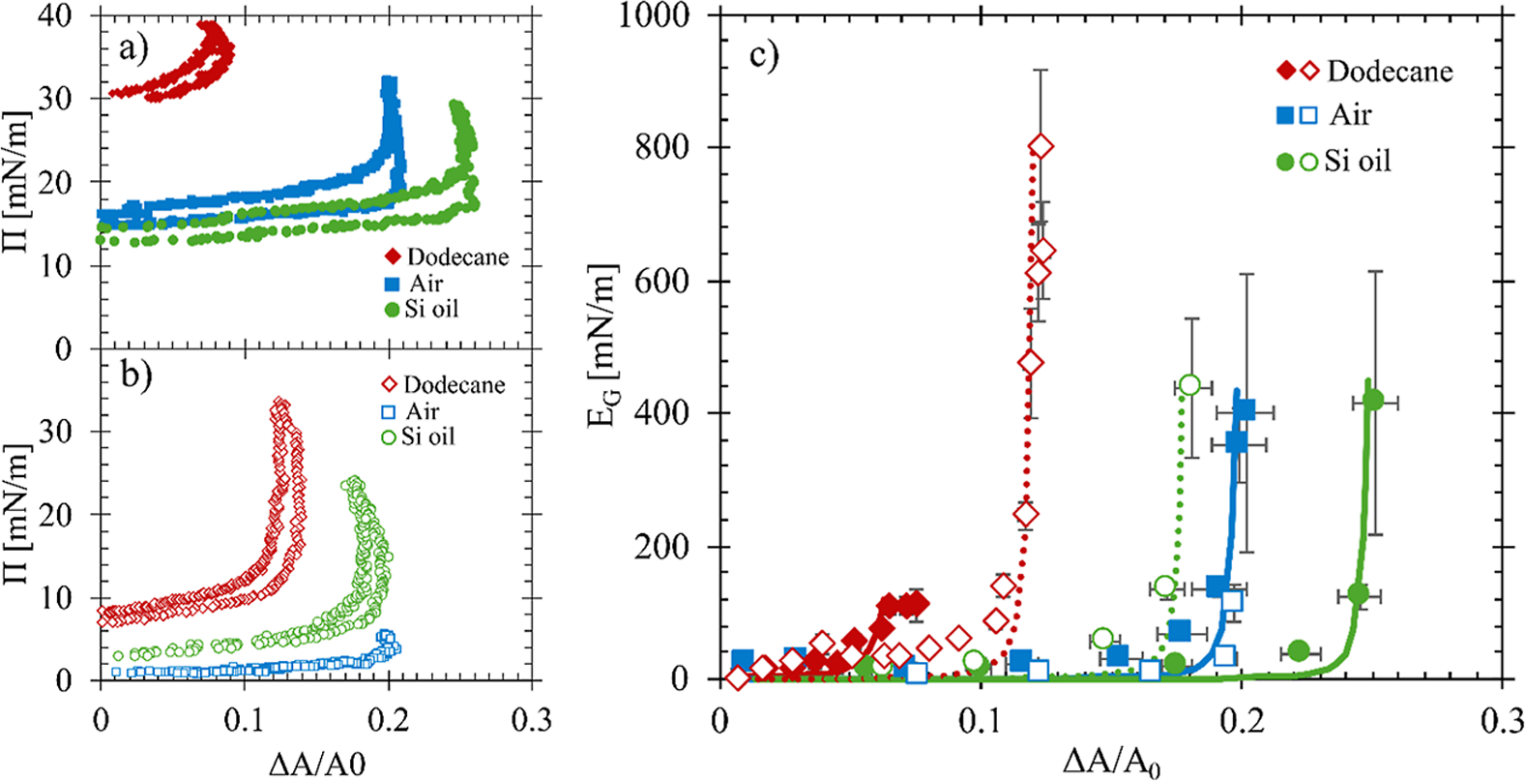
(a) Surface pressure (Π) as a function of surface area for an interfacial expansion and compression for the air/water (squares) *n*-dodecane/water (diamonds) and silicone oil/water (circles). CTAB-SiO_2_ complexes are delivered to the interface with a 1000 s adsorption time prior to the compression process. (b) Compression process of the air/water, *n*-dodecane/water, and silicone oil/water interfaces after a bulk exchange of DI water. (c) Gibbs modulus as a function of the fractional area changes for air/water (squares), silicone oil/water (circles), and *n*-dodecane/water (diamonds) interfaces. Closed symbols are for systems with the CTAB-SiO_2_ complexes present in the bulk reservoir and open symbols are after a bulk fluid exchange with DI water. Solid lines are fits to the Volmer EOS and added to guide the eye. Dotted lines are fits for the rinsed systems. For all systems, the concentration of CTAB used was 0.3 mM.

In the oil/water systems, there is a change in the direction of the slope along the surface pressure measurement that is not observed at the air/water interfaces. This change in the measured signal is a nonphysical result that is an indication of nonhomogeneous behavior at the interface during the compression cycle. The shape of the compression curves as well as the compression size required to jam the interfaces change significantly in systems that undergo the reservoir rinsing process prior to the compression cycles, as shown in Figure 4b. It is assumed that the surface-active species, CTAB, is the main contributor to the surface pressure; however, the shapes of the compression curves for each interface indicate a different interfacial response to processing due to the different hydrophobic phases. Thus, surface pressure measurements alone are insufficient to describe the particle coverage and jamming behavior at the interface. Figure 4c shows the Gibbs modulus for the interfaces shown in Figure 4a and b, with closed symbols corresponding to compressions after 1000 s of adsorption of CTAB-SiO_2_ complexes and open symbols corresponding to compressions after a bulk reservoir exchange with DI water. These mechanical measurements are needed to determine the impact of adsorbed particles to interfaces with different hydrophobic phases. Lines fit to the Volmer equation of state are added for each interface and used here to guide the eye. The horizontal error presented in Figure 4c is from the propagation of error in the optical measurement. At small Δ*A*/*A*_0_ values, the error is small and approaching the size of the symbols on the plot.

Although the surface pressure measurements suggest similar surface coverages at air/water and dodecane/water interfaces due to similar bulk surfactant concentrations, the critical area change required to jam an interface varies when changing the hydrophobic phase. At the air–water interface, jamming behavior is the same for the system exposed to CTAB-SiO_2_ complexes and the system with fresh DI water in the bulk reservoir, with a ξ_c_ of 0.20. This, however, is not observed at oil/water interfaces. With the CTAB-SiO_2_ complexes present in solution, the value for ξ_c_ on the first interfacial compression is 0.70 and 0.25 for dodecane and silicone oil, respectively. After the bulk fluid exchange with DI water, ξ_c_ increases to 0.12 at the dodecane/water interface, while for silicone oil, this value decreases to 0.18. This result indicates that, while the rinsing process has little effect on jamming behavior at air/water interfaces, changes to the aqueous fluid properties during rinsing significantly impact behavior at an oil/water interface under similar experimental conditions.

Assuming all the particles remain pinned to the interface as the interface is compressed, ξ_c_ is used to estimate the surface concentration ratio ( $\frac{\phi_{0}}{\phi_{c}}$ ) for each system as

9 $$\xi_{c}=1-\frac{\phi_{0}}{\phi_{c}}$$

where ϕ_c_ is estimated as the maximum packing fraction of 2D random close packing (ϕ_c_ = 0.86). The values for the critical area change and the corresponding surface concentration ratio from the data in Figure 4c are shown in Table 2. For hard sphere interactions, $\frac{\phi_{0}}{\phi_{c}}$ is directly related to the cross-sectional area of the particle (*A*_p_) at the interface

10 $$\phi =\frac{{NA}_{p}}{A}$$

where *A*_p_ is the cross-sectional area of the particle at the surface and *N* is the number of adsorbed particles. This cross-sectional area is controlled by the contact angle at the three-point contact line, which is evaluated from the interfacial forces between each surface as

11 $$\cos\left(\theta \right)=\frac{\gamma_{op}-\gamma_{wp}}{\gamma_{ow}}$$

where γ_op_ is the oil-particle interfacial tension and γ_wp_ is between the water and particle. This angle determines the position of the particle relative to the interfacial boundary as well as the energy of adsorption associated with particle wetting. However, changing the position and wetted area of the particle at the interface does not change the center-to-center distance between particles. Thus, the steric interactions between particles at the interface does not fully account for the differences in ξ_c_ seen when the hydrophobic phase is changed. Unlike air/water systems, particles at the interface have the potential of material entering the oil phase due to the deformation process. However, the adsorption energies for the systems used in this work are all on the order of 10^2^–10^3^ kT (eq 1), and complete removal from the interface is unlikely.

**Table 2:** Critical Area Change (ξ_c_) and Surface Concentration Ratio ( $\frac{\phi_{0}}{\phi_{c}}$ ) from the Compression of a Single Interface for the Air/Water, Dodecane/Water, and Silicone Oil/Water Systems

hydrophobic phase	critical area change (ξ_c_)	surface concentration ratio ( $\frac{\phi_{0}}{\phi_{c}}$ )
air	0.20	0.80
dodecane	0.07	0.93
dodecane after bulk exchange	0.12	0.88
silicone oil	0.25	0.75
silicone oil after bulk exchange	0.18	0.82

In addition to the physical areal coverage of particles, the jamming behavior is directly impacted by the length scale at which the particles begin to interact laterally. The effects of interparticle interactions on the interface are investigated by controlling the ionic strength of the bulk reservoir fluid and screening the electrostatic interactions in the aqueous phase. Charge screening in the aqueous phase decreases the energy barrier for particle adsorption and alters the electrostatic interactions.^18,40,58^ Following a procedure developed for air/water interfaces,^2^ CTAB-SiO_2_-coated interfaces are exposed to 100 mM NaCl salt solution and then subjected to the compression experiments. To avoid the flocculation of nanoparticles with this increased ionic strength, controlled sequential bulk reservoir exchanges were used. First, interfaces were exposed to an aqueous phase of 0.1 mM CTAB with 10 wt % SiO_2_ nanoparticles in 10 mM NaCl for 1000 s to allow for interfacial adsorption. Then, the bulk reservoir was exchanged with DI water, removing excess bulk material and leaving the interfaces coated with the irreversibly adsorbed SiO_2_ nanoparticles. The reservoir fluid was exchanged once more, replacing the DI water with 100 mM NaCl after which, the compression experiment was performed. Figure 5 shows a series of compression cycles on a single air/water (a) and dodecane water (b) interface exposed to 100 mM NaCl. Air/water measurements are from work in a previous study^2^ and is replotted here for comparison against the oil/water interfaces processed under similar experimental conditions. Figure 5c shows data for a single compression cycle of a particle-coated silicone oil/water interface exposed to 100 mM NaCl.

**Figure 5:**
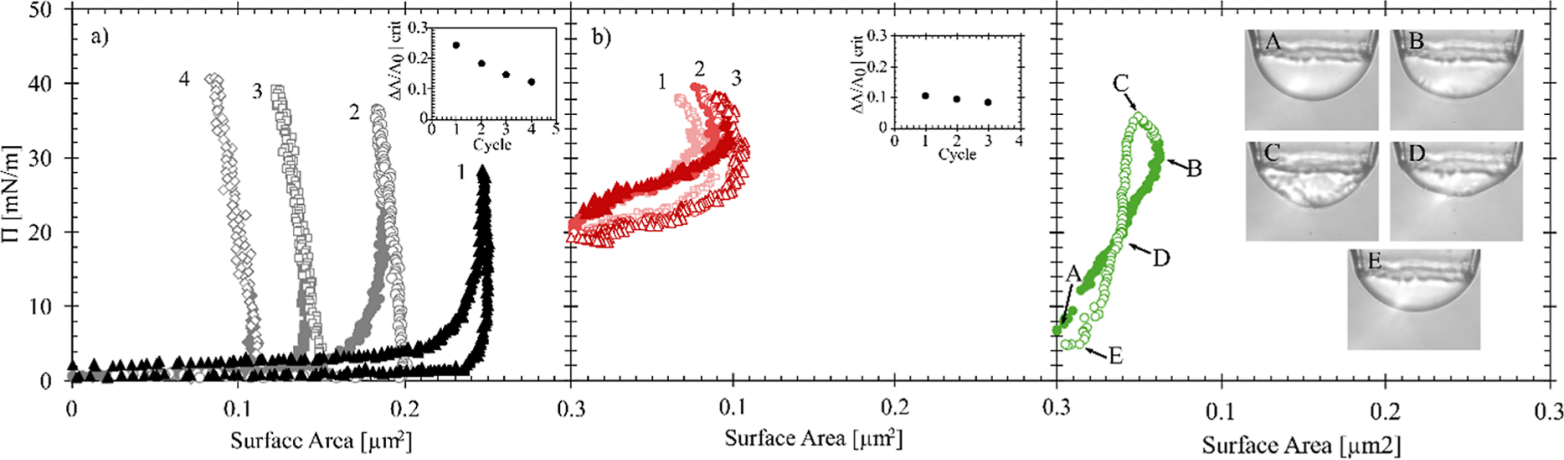
Surface pressure (Π) as a function of surface area for consecutive compression cycles of the (a) air/water, replotted with permission from Kirby et al.^2^ The concentration of CTAB used here was 0.1 mM CTAB. (b) Dodecane/water interfaces exposed to 100 mM NaCl. The insets on each plot show the critical area change required for jamming at each compression cycle. (c) A single compression and expansion cycle of a nanoparticle coated silicone oil/water interface exposed to 100 mM NaCl. The shape of the silicone oil/water interface along the compression cycle are shown with the images A–E. Open symbols represent the points at which the slopes of the compression curves change direction. The concentration of CTAB used was 0.3 mM for both dodecane/water and silicone oil/water systems.

Significant changes in the surface pressure response as well as the difference in critical compression size between cycles are observed at the air/water interface. With increased ionic strength in the aqueous phase, the critical surface area is more sensitive to the experimental conditions and deformation history of the interface, and ξ_c_ decreases with each compression cycle. On the second compression, the direction of the slope in the surface pressure response is reversed and is a response indicative of nonhomogenous behavior at the interface. This behavior is interpreted as the formation of a percolated network of particles at the interface,^2^ where the compression process forces absorbed particles to aggregate and irreversibly change the interfacial structure.^40^ At these high salt concentrations, the large interfacial compression at the air/water interfaces forces an irreversible change to the interfacial structure, which is interpreted here as a forced aggregation of adsorbed particles due to the deformation performed.

At the dodecane/water interface, there is a shift in the critical surface area that follows a trend similar to that of air/water but with much smaller changes between each compression cycle, as shown in Figure 5b. Additionally, irreversible changes to the structure are observed on the first compression, as evidenced by the change in direction of the slope of the surface pressure measurement, indicating that particles at the dodecane/water interface are aggregating prior to any interfacial processing. With increased ionic strength at a silicone oil/water interface, shown in Figure 5c, the silicone oil/water system demonstrates solid-like interfacial behavior during the compression process. There is an immediate, sharp increase in the surface pressure with a decreasing surface area, followed by a change in slope of the compression curve and an interfacial collapse. Images of the interface during the compression and expansion of the interface are shown with the inset images and are labeled A–E to indicate the points along the compression cycle of the interface that correspond to the observed interfacial behavior. A video of the compression and expansion of a silicone oil/water interface exposed to a high ionic strength aqueous phase is shown in the S2 (video) of the Supporting Information. As the interface is compressed, visible wrinkles begin to form (B), while further compression leads to buckling and collapse of the interface (C, D). Expanding the interface back to its initial surface area restores the shape of the interface (E), but the initial surface pressure is not recovered after this interfacial collapse. This behavior is indictive of the development of a rigid, solid-like network of particles at the interface^17^ and was only observed at particle-coated silicone oil/water interfaces exposed to 100 mM NaCl in the aqueous phase. Enhanced surface aggregation of charged nanoparticles at oil/water interfaces has been observed in the literature,^4^ where the nature of the oil surface lends itself to additional attractive forces between particles. This is in good agreement with what has been observed in our systems where the oil/water interfaces demonstrated behavior that suggests the formation of aggregates at the interface before any interfacial compressions occur.

Results from Figures 4 and 5 show data for a single experiment on each type of interface to demonstrate the different interfacial responses due to changes in the nature of the hydrophobic fluid. Using the same aqueous conditions from Figure 4, additional experiments at both dodecane/water and silicone oil/water interfaces show variation in the interfacial responses between repeated experiments, as shown in Figure S3 of the Supporting Information. As each curve corresponds to the first compression of a single interface, the variation in both the shapes of these curves as well as the critical areal change (ξ_c_) is likely due to nonhomogenous interfacial behavior and could further indicate the formation of a particulate network of aggregates at these oil/water interfaces prior to any interfacial processing. These results demonstrate that the particle interactions are more complex due to the presence of the oil, the nature of the hydrophobic phase directly impacts the interparticle forces at the interface, and these results indicate the interactions between particles through the oil have a significant effect on the particle jamming behavior. Multiple phenomena may explain the differences in observed behavior due to the nonpolar phase. It is important, however, to note that the CTAB-SiO_2_ complexes adsorb from the aqueous phase in all cases, and significant differences in particle–particle interactions through the aqueous phase are not expected. Rather, differences in the jamming behavior and particle interactions at the interface are expected to arise from environmental changes due to the nonpolar phase. These include changes in the wetting and contact angle (eq 11) that impact particle position at the interface. Additionally, particle–particle interactions through the nonpolar phase would arise form variations in the permittivity of the oil and polarization state of the particle surface in the nonpolar phase.^59,60^ While these interactions are not well understood, they become important in concentrated colloidal systems like the ones formed at these fluid interfaces.^4^

## Conclusions

While the particle behaviors at air/water and oil/water interfaces show general similarities, the oil/water systems are shown to be more complex. This is likely due to additional interactions between particles at the interface that have been introduced by the oil. By altering the nature of the hydrophobic fluid, the mechanical properties of particle jamming at the interface are significantly altered. The phenomenon of particle jamming is controlled by the areal coverage as well as the interaction of particles at the interface, and both can be tuned with the composition of the hydrophobic phase. The amount of material at the interface is determined by the adsorption of the surfactant, which is dependent on the surfactant concentration as well as the nature of the hydrophobic phase. With isotherm information about the nature of surfactant adsorption, the number of particles delivered to the interface can be controlled. By modification of the surface properties with additives such as surfactants, the wetting properties and interparticle interactions can be adjusted. We also observe what appears to be a change in the particle–particle lateral interactions through the nonpolar phase. While the electrostatic interactions through the aqueous phase are recognized to control lateral interactions, here we are seeing significant changes in the interactions through the nonpolar phase causing drastic differences in shifting from air/water to oil/water phases with ramifications in designing Pickering emulsions rather than foams. With the identification of key tools to control interfacial behavior, this work provides an important framework for understanding methods for the design and processing of particle-coated interfaces with desired mechanical properties.

## Supplementary Material





## Notes

Zeta potential measurements of bulk surfactant-nanoparticle aqueous solutions, a video showing the interfacial compression of a particle-coated silicone oil/water interface exposed to 100 mM NaCl, and compression behavior of dodecane/water and silicone oil/water interfaces between different experiments under the same conditions (PDF)
Videos files for S1 and S2 (AVI)
