# Supplementary material for: Effects of Hydrophobic Phase Properties on Controlling Nanoparticle Jamming at Oil/Water and Air/Water Interfaces
Source: Langmuir. 2025 Aug 7;41(32):21415–24. doi: 10.1021/acs.langmuir.5c01946 (PMC12368986; doi:10.1021/acs.langmuir.5c01946)
Supplement: Supplementary file 1 [file la5c01946_si_001.pdf]

Supporting information – Effects of hydrophobic phase properties on controlling nanoparticle jamming at oil/water and air/water interfaces.

Olivia M. Haider[1] and Lynn. M. Walker[2,*]

[1] Department of Chemical Engineering, Carnegie Mellon University, Pittsburgh, Pennsylvania, 15213, United States

[2] Department of Chemical Engineering and Materials Science, University of Minnesota, Minneapolis, Minnesota, 55455, United States

*corresponding author: lmwalker@umn.edu

Details:

Number of pages: 3

Number of figures: 2

Number of videos: 1

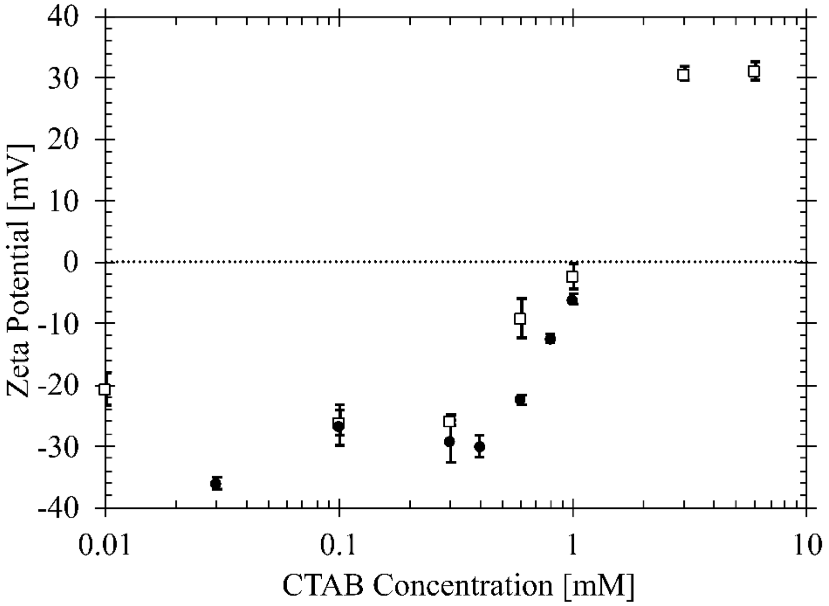

S1. Zeta potential measurements of 1 wt% dispersions of Ludox TMA nanoparticles at varied concentrations of CTAB in 10 mM NaCl. Closed symbols are measurements using the Ludox TMA used as received from Sigma (Saint Louis, MO), while open symbols are measurements from a previous air/water interfacial study,[1] replotted with permission and added for comparison. The slight differences in zeta potential between each study can be attributed to the different lot numbers of the Ludox TMA samples.

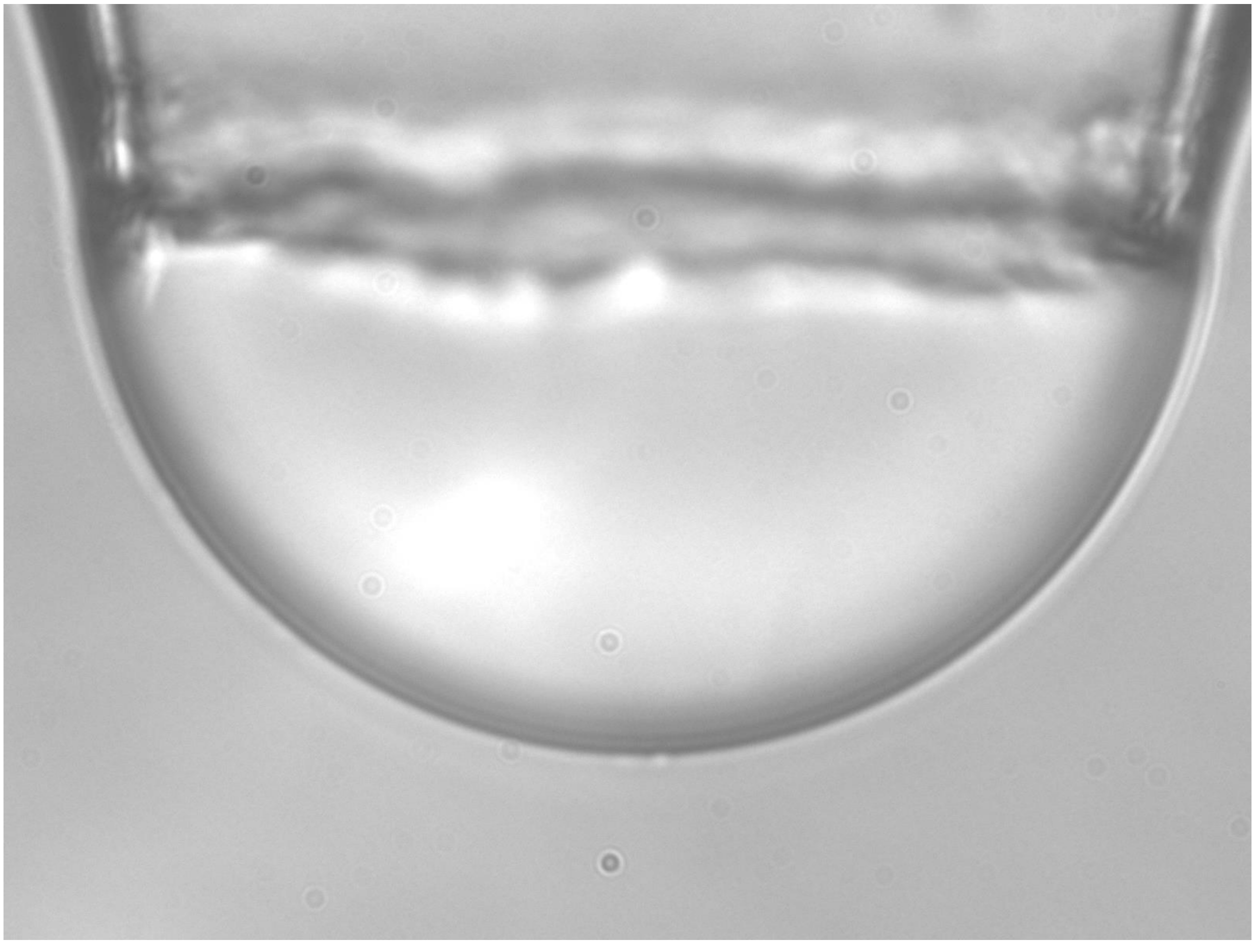

S.2 Video during an interfacial compression at 0.2 x speed with silica nanoparticles pinned at a silicone oil/water interface exposed to 100 mM NaCl. Silicone oil is located inside the capillary, with a capillary tip of radius R_c = 42 µm. The aqueous fluid containing 100 mM NaCl is located outside the capillary in the bulk reservoir. The direction of gravity is into the page. The observed wrinkling and buckling of the interface upon compression indicates a jammed interface that is compressed to the point of interfacial failure.

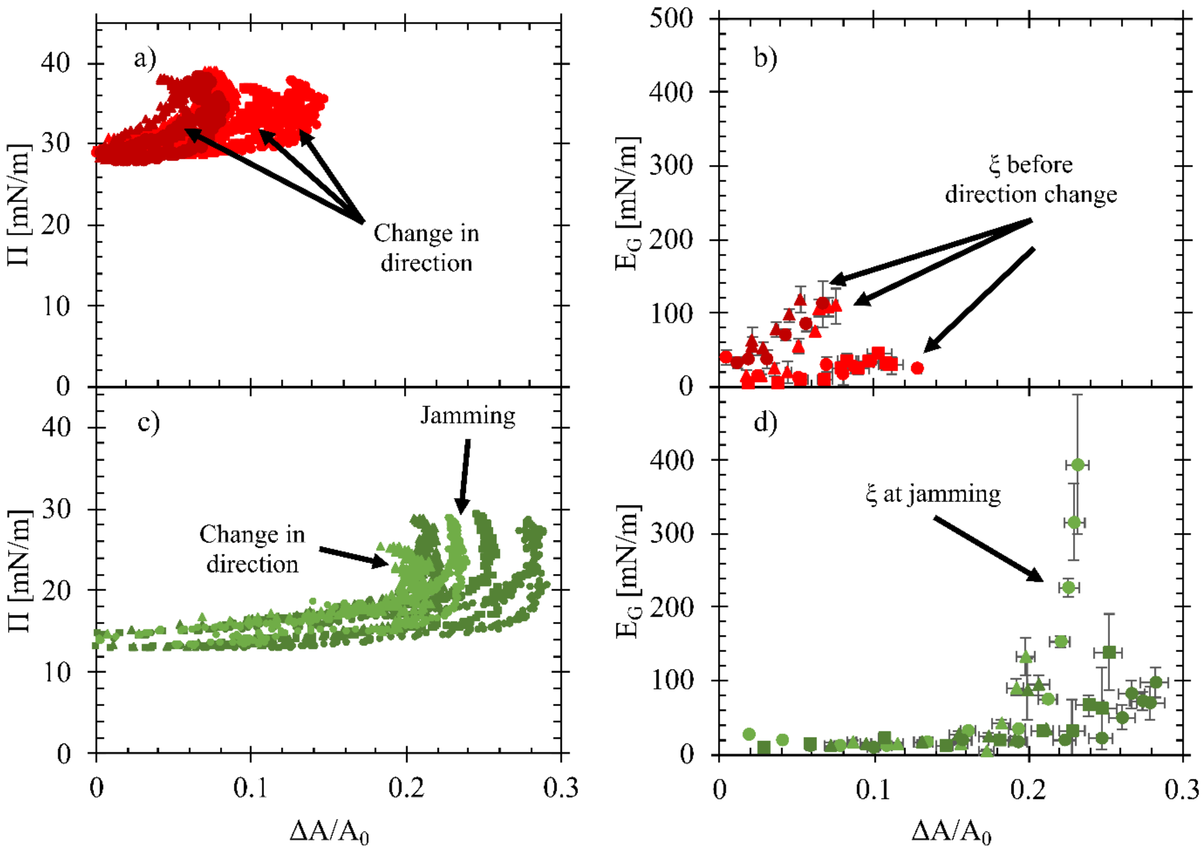

S3. Surface pressure (a) and Gibbs modulus (b) for compressions to dodecane/water interfaces exposed to 0.3 mM CTAB with 10 wt % SiO_2 in 10 mM NaCl for a 1000 seconds adsorption time. Each loop corresponds to the first compression of an individual interface. The surface pressure and Gibbs modulus for the first compression of silicone oil/water interfaces are shown in (c) and (d) respectively. Corresponding colors indicate replicate experiments with aqueous solutions prepared on the same day. Arrows indicate the position on the compression loops at which the surface pressure increases sharply or the slope of the curve changes direction.

References:

(1) Kirby, S. M.; Anna, S. L.; Walker, L. M. Effect of Surfactant Tail Length and Ionic Strength on the Interfacial Properties of Nanoparticle–Surfactant Complexes. *Soft Matter* **2018**, *14* (1), 112–123. https://doi.org/10.1039/C7SM01806A.
